# Exploring Inequality Through Service Learning in Higher Education: A Bibliometric Review Study

**DOI:** 10.3389/fpsyg.2022.826341

**Published:** 2022-03-11

**Authors:** Nazaret Martínez-Heredia, Silvia Corral-Robles, Gracia González-Gijón, Micaela Sánchez-Martín

**Affiliations:** ^1^Department of Pedagogy, Faculty of Education, University of Granada, Granada, Spain; ^2^Department of Didactics of Language and Literature, Faculty of Education, University of Granada, Granada, Spain; ^3^Department of Research Methods and Diagnosis in Education, Faculty of Education, University of Murcia, Murcia, Spain

**Keywords:** bibliometric review study, service-learning, inequality, higher education, social justice

## Abstract

Service learning (S-L) is an innovative methodology, which is extensively known worldwide. The implementation of this methodology involves classroom learning and real practice. It is based on a cooperative methodology, integrating community service and learning in a connected way. Its main strength lies in its great potential as a transformative social movement to reduce inequality. The main aim of this study was to understand and describe the field of S-L and inequality in higher education through a bibliometric analysis. A descriptive, retrospective, and cross-sectional methodology is used to describe the information obtained from the 20 references on the topic registered in the Scopus and Web of Science (WoS) databases using a mixed methodology. The quantitative and qualitative results show that most of the publications are concentrated in 2016, with the United States being the country with the largest amount of scientific production on this subject. It is also worth noting that most of the authors reported this approach as a powerful tool to develop consciousness, commitment, and responsibility toward inequality and social problems.

## Introduction

University education presents multiple and diverse scenarios in which it is complicate to stick to old approaches (Pinto et al., 2016). Times have changed. At present, university students demand practical training as they know the difficulties they will face in their professional career. In the attempt to fulfill this need, the European Higher Education Area (EHEA) states that any training model should be focused on the acquisition of competences, rather than the acquisition of knowledge. The combination of skills, knowledge, and attitudes makes an individual efficient enough to face difficulties such as inequality, social exclusion, and discrimination within a specific context (López-Ruiz, 2011).

This change of approach implies a profound renewal of the teaching methodologies traditionally used in the university (Parra-González et al., 2020). Thus, it is necessary to use innovative teaching proposals that help to integrate professional and socially responsible practice. Martínez (2008) considered that “it is necessary that the training model of each university promotes in its practice teaching, learning and research, and working spaces, situations that imply community involvement and make possible the improvement of living conditions in the territory” (p. 7).

Under this premise, service learning (S-L) is an innovative methodology, whose implementation involves classroom learning and real practice. It is based on a cooperative methodology, integrating community service and learning in a connected way. Its main strength lies in its great potential as a transformative social movement to reduce inequality from an interdisciplinary perspective (Martínez and Folgueiras, 2015).

In this context, the term “inequality” can be understood as social disparity, that is, an unfair situation in which some people have more rights or better opportunities than other people. This definition is intrinsically related to the experiences that can be promoted with the implementation of the S-L methodology as a pedagogy designed to engage students in the firsthand experiences of global social issues, including aspects such as inequality (Billig, 2017).

The very act of immersing university students into underserved communities provides them the opportunity to develop empathetic understanding and concern about the causes of inequality. This impacts on the emergence of solidary attitudes and cooperative responsibility for the achievement of common objectives, favoring the establishment of more cohesive, empathetic, affective, and communicative bonds with their vision of a global society (Freire, 1970; Endres and Gould, 2009; Cipolle, 2010; Yoder Clark, 2010; Ruiz-Montero et al., 2021).

The variety of fields of knowledge in this area of study has contributed to the diversity and multiplicity of research work. However, preparing a study on inequality through S-L in higher education is linked to different and complex frameworks due to its interdisciplinary approach. This makes difficult for researchers to define concepts and chart the right research path.

The main aim of this study was to understand and describe the field of S-L and inequality in higher education through a bibliometric analysis. The results in the bibliometric analysis will allow us to study the topic on two of the most well-known and prestigious databases, such as Web of Science (WoS) and Scopus. Moreover, following this bibliometric analysis will avoid bias and search selection problems. A descriptive, retrospective, and cross-sectional methodology is used to describe the information registered in the Scopus and WoS databases using a mixed methodology, with the first part of the results based on a quantitative analysis and the second part based on a qualitative analysis.

This bibliometric review starts from the following research question formulated according to the so-called PICO format: What is the contribution of bibliometric analysis to the review and development of theoretical literature on inequality through S-L in higher education?

Population (P): higher education users

Intervention (I)/Exposure (E): S-L programs

Comparison (C): not applicable

Outcome (O): contribution of bibliometric analysis to the review and development of knowledge on inequality

Studies (S): all study designs, except case report studies, reviews, case series, conference papers or posters, opinion pieces, editorials, commentaries, or policy papers

Thus, seven specific questions arise from the general question:

RQ1. What is the importance of bibliometric analysis in defining the theoretical framework for the inequality through S-L in higher education?

RQ2. What diachronic and personal productivity is formed by the publications and citations in inequality through S-L in higher education?

RQ3. Who are the most cited authors in the field of inequality through S-L in higher education?

RQ4. Which research documents are cited the most frequently by authors in the field of inequality through S-L in higher education?

RQ5. What are the most important research institutions concerning the production of research papers in inequality through S-L in higher education?

RQ6. What are the most important countries concerning the production of research papers in inequality through S-L in higher education?

RQ7. Which keywords do authors on inequality through S-L in higher education use the most frequently?

From the research questions posed, we set out the following specific objectives that would be attained through a quantitative analysis: (I) to find out the diachronic productivity and compliance with Price’s Law; (II) to analyze authors and specialized sources by checking Lotka’s Law; (III) to analyze Bradford’s Law or the law of dispersion; and (IV) to analyze the most relevant impact indicators: (a) type of document, (b) internationalization of research or country of publication, (c) language, (d) authorship affiliation, (e) most cited reference journals, (f) documents with the highest impact, (g) authors with the highest impact, and (h) bibliometric mapping of keywords through content co-occurrence analysis. Similarly, from a qualitative point of view, the aim was (V) to find out about the following: (i) the methodological design, (j) the research instruments, (k) the sample of these studies, (l) the objectives, and finally (m) the results.

## Methodology

This article is a bibliometric review characterized by the systematization process of searching, selecting, and analyzing the literature to support the substantiation and consolidation of a quantitative description of the information obtained (Expósito-López and Olmedo-Moreno, 2020; Martínez-Heredia et al., 2021; Panta-Carranza et al., 2021). This type of articles allows researchers to check the contributions already made in this field of study (França et al., 2021; Giménez Lozano et al., 2021).

To describe and understand inequality through S-L in higher education, the search process was carried out following the protocol outlined in the PRISMA statement (Preferred Reporting Items for Systematic reviews and Meta-Analyses, 2020). This statement is followed in order to systematize the evidence found in an organized way, through the rigorous use of a series of methods and techniques for planning, searching, and presenting the information to promote its replicability.

The search was conducted in WoS and Scopus databases. A 15-year period was established, considering that, in the past 5 years, the S-L methodology has had a notable growth in higher education. The inclusion criteria were as follows: (I) original research articles on S-L in higher education, books, and book chapters; (II) published between January 2006 and September 2021; (III) qualitative, quantitative, or mixed studies; (IV) articles in English or Spanish; (V) availability of full text; and (VI) studies that include the descriptors analyzed in the title, abstract, and keywords. The exclusion criteria established for studies were as follows: (I) studies published in any language other than Spanish or English; (II) other types of studies (e.g., reviews, case series, conference papers or posters, opinion articles, editorials, commentaries, or political documents, among others); and (III) studies that do not address the subject matter analyzed.

Search strategies were established according to the main objective of the article. In the first phase, descriptors and Boolean connectors were defined for the search in the corresponding databases (“Service-learning” and “Inequality,” “Service-learning” and “Higher education,” “Inequality” and “Higher education”). By doing so, a specific database, whose validity of use is defined by authors such as Beltrán (2005); Urrútia and Bonfill (2010), and Expósito-López and Olmedo-Moreno (2020) was created.

The results obtained included clearly ineligible studies; therefore, the main researchers performed a rapid screening of the studies identified in the bibliometric searches by reviewing titles and abstracts and eliminating those not clearly related to the research topic. Subsequently, a panel of two reviewers independently and blindly determined compliance with the inclusion/exclusion criteria of the studies resulting from the first screening by reviewing the full article. In cases where there was disagreement, a discussion was held between the reviewers in search of consensus, and when disagreement persisted on any aspect, a third reviewer was involved.

Before starting the data extraction process, a coding manual and a protocol for recording the data have been drawn up in which the variables selected to obtain the information are determined: (I) outcome: studies on inequality and S-L in higher education; (II) context: country of implementation; (III) methodological: study design; and (IV) extrinsic aspects: journal, author, language, year of publication, professional affiliation of authors, co-occurrence of content, and impact. Table 1 shows the variables analyzed and the criteria considered for their analysis.

**TABLE 1:** Data extraction variables.

Variable	Inclusion criteria
Year of publication	Last 15 years
Type of document	Research articles on the topic, books and book chapters
Affiliation	All authors affiliations are considered
Language	English and Spanish
References with the greatest impact	All citations are considered
Internationalization of research	All countries are considered
Publication authorship	All authors are considered
Reference journals	All journals indexed in WoS and SCOPUS databases are considered
Co-occurrence of content	Title, abstract and keywords
Study design	Qualitative, quantitative and mixed designs are considered.

**FIGURE 1:**
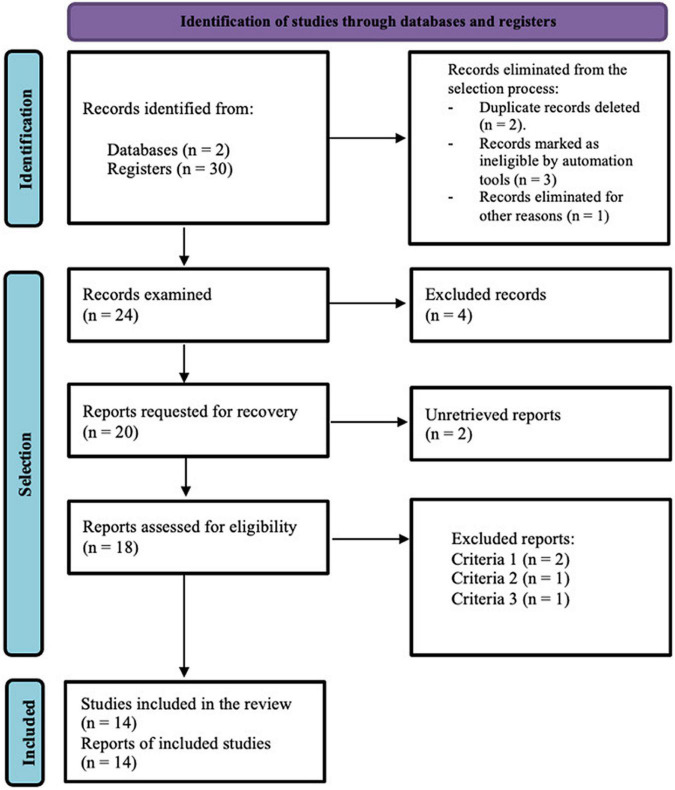
Descriptive flowchart of the sampling process (PRISMA 2020). Source: Adapted from Page et al. (2021).

In the third phase, the results are synthesized, as shown in Figure 1.

## Results and Discussion

This section contributes to answer the main objective, i.e., to understand and describe the field of S-L and inequality in higher education through a bibliometric analysis. Therefore, this section is an attempt to answer the specific objectives presented earlier:

### Objective 1: To Find Out the Diachronic Productivity and Compliance With the Price’s Law

**FIGURE 2:**
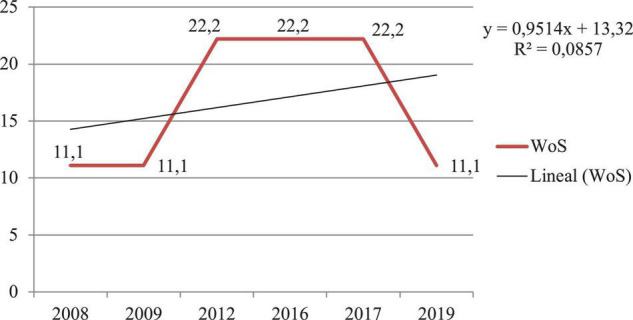
Diachronic production of scientific production indexed in the Web of Science (WoS) database.

**FIGURE 3:**
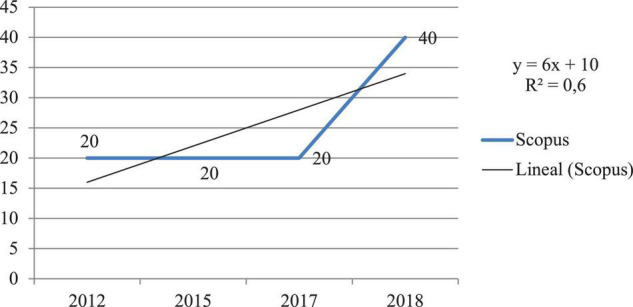
Diachronic production of scientific output indexed in the Scopus database.

Considering the diachronic productivity and the year of publication variable, in both databases, it can be seen that there has been exponential growth in the research topic under study; however, in the WoS database, it is highlighted that the highest scientific productivity has occurred during 2012, 2016, and 2017 (*n* = 2; 22.2%) and in Scopus during 2012 (*n* = 2; 40%). The WoS database has the highest scientific production during the number of years studied. Figures 2, 3 show the results analyzed by year and database.

Checking Price’s Law, based on the exponential growth of scientific information, it can be noted that this theory is fulfilled as the research is in phase 3 of linear growth. The rate of increase remains constant or independent of the size of the system (Price, 1986). The amount of production and its proportion percentages remain very stable, about 1–2 publications and 20–40% in WoS and 11–22% in Scopus.

### Objective 2: To Analyze Authors and Specialized Sources by Checking Lotka’s Law

**FIGURE 4:**
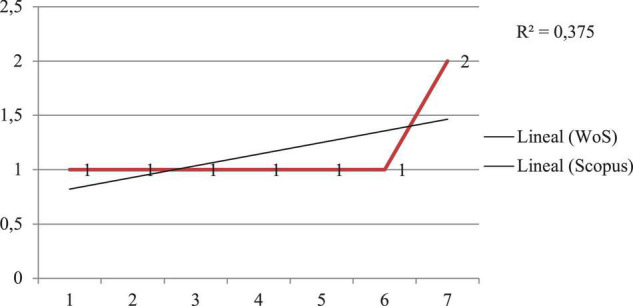
Production of scientific output indexed in the WoS and Scopus databases.

Lotka’s law or productivity law refers to the fact that a small proportion of authors is responsible for the majority of scientific papers, so that when the number of papers on a given topic increases, the number of authors decreases (Urbizagástegui, 2005). Figure 4 shows that the correlation between the lower number of authors and the higher number of scientific outputs in both databases is positive. Pearson’s correlation coefficient in both databases is *r* = 375, which means that there is a linear correlation with a perfect positive slope, assuming absolute determination between both variables.

### Objective 3: To Analyze Bradford’s Law or the Law of Dispersion

**FIGURE 5:**
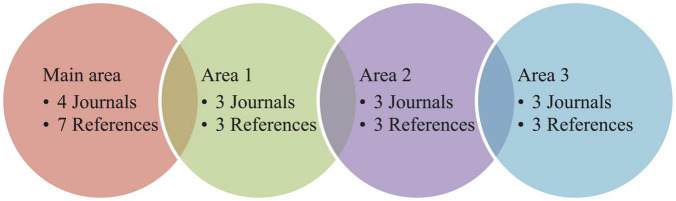
Dispersion of scientific production indexed in the WoS and Scopus databases.

Bradford’s Law or law of dispersion refers to the fact that the majority of articles on a given subject could be published by certain specialized journals, together with certain general or dispersion journals. There exists a relationship between the articles published and the journals in a given area; thus, there is a similar number of articles and journals grouped in different areas of dispersion (Urbizagástegui, 2016). In total, there are 13 journals and 16 references distributed in four areas, and we observed that the main area included 4 journals and 7 references. This can be seen in Figure 5 However, in the other three areas, there is a similar number of journals and references.

### Objective 4: To Analyze the Most Relevant Impact Indicators

(a) Type of document

Referring to the type of document, in both databases, the “scientific article” occupies the highest percentage of reference (*n* = 3–60% in Scopus and *n* = 7–77.8% in WoS). Similarly, in Scopus, we found “book chapters” with only 40% (*n* = 2). However, in the WoS database, the “book” is the type of document that can be highlighted (*n* = 2; 22.2%).

(b) Internationalization of research or country of publication

With regard to the country of publication, the United States stands out with 60% (*n* = 3) in Scopus and 66.67% (*n* = 6) in WoS, with a large number of publications compared with the rest of the countries. Similarly, in the Scopus database, Canada has 2 publications (*n* = 2; 40%) and, in the WoS database, Spain, England, and Ecuador have 1 publication each (*n* = 1; 11.11%).

(c) Language

Regarding the language variable in scientific production, English is the only language used for the publication of these references in both databases.

(d) Authorship affiliation

The affiliation with the highest number of authors in both databases is the University of Kentucky, with 20% in the Scopus database and 22.2% in the WoS database (out of the 15 institutions found) This can be seen in Table 2 and Table 3.

**TABLE 2:** Number of documents by affiliation found in the Scopus database.

Affiliation	Number	Percentage
University of Kentucky	1	20
King’s University College at Western University Canada	1	20
DePaul University	1	20
Virginia Polytechnic Institute and State University	1	20
University of Alberta	1	20

**TABLE 3:** Number of papers by affiliation found in WoS database.

Affiliation	Number	Percentage
DePaul University	2	22.2
Beck Res Initiat Women Gender Community	1	11.1
Calvin Coll	2	22.2
Durban University of Technology	1	11.1
Indiana State University	2	22.2
Ithaca Coll	1	11.1
Univ. Europea	1	11.1
University of Kentucky	2	22.2
Virginia Polytechnic Institute State University	2	22.2
Univ. San Francisco Quito Usfq	1	11.1

(e) Most cited reference journals

The journals with the highest production in Scopus database regarding the topic under analysis are *Administration and Society*, *Citizenship Teaching and Learning*, *Handbook of Engaged Sustainability*, *Journal of Geography*, and *Journal of Transformative Education*.

In contrast, the journals with the highest production are *Administration and Society*, *Advances in Service-Learning Research*, *Innovations in Higher Education Teaching and Learning*, *Journal of Geography*, *Journal for New Generation Sciences*, *Journal of College Student Development*, *Journal of Transformative Education*, and *Learning and Teaching the International Journal of Higher Education in the Social Sciences*.

The most cited reference journals that coincide in both databases are *Administration Society*, *Journal of Geography*, and *Journal of Transformative Education*, with 1 reference published in each journal indexed in each of the databases analyzed.

(f) Articles with the highest impact

Regarding the variable “articles with the highest impact,” we found that the article “Service-learning: critical traditions and geographic pedagogy,” from the *Journal of Geography* is the most cited in the Scopus database with a total of 10 citations, followed by the article “A proposed workshop curriculum for students to responsibly engage cultural conflict in community-based service-learning” from the *Journal of Transformative Education* with 6 citations. This is followed by the article “A proposed workshop curriculum for students to responsibly engage cultural conflict in community-based service-learning” from the *Journal of Transformative Education* with 6 citations. In contrast, in the WoS database, the first most cited article “The relationships between service-learning, social justice, multicultural competence, and civic engagement” has 125 citations and the second “Service-learning: critical traditions and geographic pedagogy” coincides with the first in the Scopus database, but in this case with 9 citations This can be seen in (Table 4 and Table 5).

**TABLE 4:** Most cited authors in Scopus regarding the topic of this research.

Title	References	Journals	Citations
Service-Learning: Critical Traditions and Geographic Pedagogy	Grabbatin and Fickey, 2012	Journal of Geography	10
A Proposed Workshop Curriculum for Students to Responsibly Engage Cultural Conflict in Community-Based Service Learning	Tharp, 2012	Journal of Transformative Education	6
Close encounters of the other kind: Ethical relationship formation and international service-learning education	Larkin, 2015	Citizenship Teaching and Learning	3
Administrative Law and Service Learning: Clients, Repetition, and Race	Sterett et al., 2017	Administration and Society	2
Sustainable higher education teaching approaches	Krogman and Bergstrom, 2018	Handbook of Engaged Sustainability	1

**TABLE 5:** Most cited authors in WoS regarding the topic of this research.

Title	References	Journals	Citations
The relationships between service-learning, social justice, multicultural competence, and civic engagement	Einfeld and Collins, 2008	Journal of College Student Development	125
Service-Learning: Critical Traditions and Geographic Pedagogy	Grabbatin and Fickey, 2012	Journal of Geography	9
A Proposed Workshop Curriculum for Students to Responsibly Engage Cultural Conflict in Community-Based Service Learning	Tharp, 2012	Journal of Transformative Education	4
Administrative Law and Service Learning: Clients, Repetition, and Race	Sterett et al., 2017	Administration and Society	3
Reinforcing Paternalism? The Need for a Social Justice Approach to Prepare Students for Community Engagement at Universities Technology	Grobbelaar et al., 2017	Journal for New Generation Sciences	2
DISRUPTIVE PRACTICES Advancing Social Justice Through Feminist Community Based Service-Learning in Higher Education	Catlett and Proweller, 2016	Service-Learning to Advance Social Justice in a Time of Radical Inequality	2
What History is good for Service-learning and studying the past	Smith, 2009	Learning and Teaching-The International Journal of Higher Education in The Social	1

(g) Authors with the highest production

The authors who coincide in both databases are DuPuis, N., Fickey, A., Grabbatin, B. Hubbard, F.G., Sterett, S., and Tharp, D.S. The author with the highest production is Tharp, D.S., with 1 (10%) reference in the Scopus database and 2 (9.6%) references in the WoS database (Table 6 and Table 7).

**TABLE 6:** Authors with the highest scientific production in Scopus database.

Authors	Number	Percentage
Bergstrom, A	1	10
DuPuis, N	1	10
Fickey, A	1	10
Grabbatin, B	1	10
Hubbard, F. G	1	10
Krogman, N. T	1	10
Larkin, A	1	10
Sterett, S	1	10
Tharp, D. S	1	10

**TABLE 7:** Authors with the highest scientific production in the WoS database.

Authors	Number	Percentage
Baena, V	1	4.8
Fickey, A	1	4.8
Maistry, S	1	4.8
Ramia, N	1	4.8
Catlett, B	1	4.8
Hubbard, F	1	4.8
Mattera, M	1	4.8
Smith, M	1	4.8
Collins, D	1	4.8
Grabbatin, B	1	4.8
Napier, C	1	4.8
Sterett, S	1	4.8
Diaz, K	1	4.8
Grobbelaar, H	1	4.8
Pinto, A	1	4.8
Tharp, D. S	2	9.6
Dupuis, N	1	4.8
Hubbard, F	1	4.8
Proweller, A	1	4.8
Einfeld, A	1	4.8

(h) Content co-occurrence analysis

**FIGURE 6:**
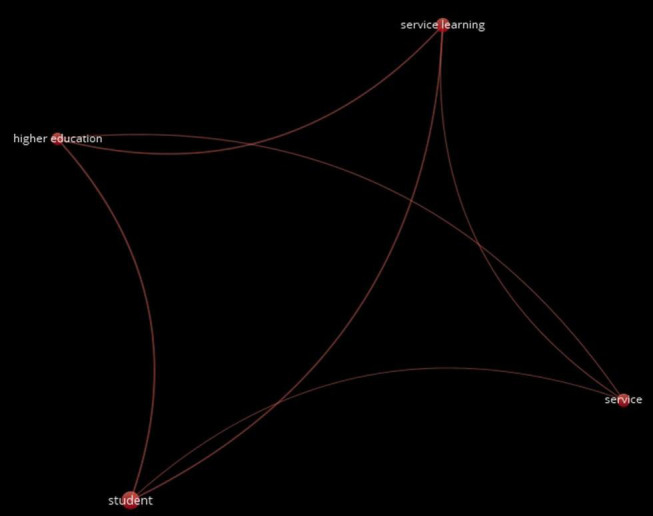
General analysis of co-occurrences.

The content co-occurrence analysis applied to the title, abstract, and keywords of the scientific production analyzed determined the existence of a thematic cluster (denoted in red), as can be seen in Figure 6. This analysis allowed us to ascertain that research on S-L from equality in higher education focuses on the intervention developed with university students (Figure 6).

So far, this research has focused on analyzing the scientific output published on the definition of the theoretical framework for inequality through S-L in higher education considering the indicators of production (diachronic and personal productivity), dispersion (correlation between authors and articles), and impact (type of document, country of publication, language, author affiliation, publications, documents with greater impact, authors with greater production, and a bibliometric map) in the WoS and Scopus database an extremely important information for future research directions.

Nonetheless, the quantitative approach of this study does not allow us to explore the content of the research analyzed; therefore, a qualitative approach has also been applied. The qualitative approach assists with the understanding of the development of an organized body of knowledge (Johnson et al., 2020). Thus, from a qualitative point of view, the fifth aim was to find out about the methodological design, the information collection instruments, the sample of these studies, the objectives, and, finally, the results.

In Supplementary Table 1, we can see the most relevant information of the contributions that have been analyzed according to the abovementioned aspects.

In Supplementary Table 1, to respond to the fifth specific objective, the documents have been analyzed according to the following five aspects:

(i) To find out about the methodological design of the works analyzed

Regarding the type of study of the documents analyzed, it can be noted that most of them are located within the qualitative paradigm as the key aspect that all the documents share is the methodology implemented, the S-L method. The qualitative approach and, therefore, the qualitative research instruments are more commonly used due to the flexibility of this method in how students and teachers assess the learning process and outcomes (Ruiz-Montero et al., 2021).

As mentioned earlier, SL is an active and experiential pedagogical methodology in which a service is provided to the community while promoting the learning of the contents (Celio et al., 2011; Asghar and Rowe, 2017). On the one hand, students learn about inequality by being in contact with the social structures that surround them, and on the other hand, society obtains their commitment and collaboration to meet a specific need (Capella et al., 2014).

(j) To know about the research instruments of the studies

Due to the social nature of these S-L experiences, they tend to be documented through qualitative research instruments. In the studies analyzed, we found some key qualitative research instruments such as participant observation, semi-structured interviews, structured interviews, focus groups, or weekly journals. Only one of the studies used a mixed method and another one a quantitative approach using a Likert scale survey.

(k) To find out about the sample chosen in the different works under analysis

It can be clearly seen the interdisciplinarity found in the different studies as the S-L method has been implemented in a great number of areas. For instance, SL has been implemented in a history honors course with 16 students at a private liberal arts college in the northeastern United States (Smith, 2009) and in a geography course with students from the Appalachian course of the department (Grabbatin and Fickey, 2012). Also, in a group of Universities of Technology students (Grobbelaar et al., 2017), students from a private liberal arts university in Quito, Ecuador (Ramia and Díaz, 2019), among others.

This shows the flexibility and adaptability of SL to different areas as it has a wide variety of choices in terms of both the service experience and the learning outcomes. This is of great help for teachers who look for a powerful experience regarding important topics such as social justice or inequality to be developed in their area of study (Chiva et al., 2016). One of the key aspects that continue to challenge university teachers is the difficulty of engaging students in ways that respond to the inequalities, and then practicing SL is a way to attract the students to navigate the challenging terrain of socioeconomic, racial, ethnic, and cultural difference (Andreotti, 2006; Jorgenson and Shultz, 2012).

Moreover, the aim (l) was to find out about the objectives set out in the different works under analysis which revolve around the discussion and reflection on the S-L experiences of students from different disciplines.

Some examples of the objectives set are as follows:

*- “The aim of this paper is to describe teaching approaches that help students understand how people, problems, and ecological conditions are interconnected being service-learning an example of the teaching approaches presented” (Krogman and Bergstrom, 2018, p.1).*

*- “The aim of this paper is to examine how participants in a long-term service-learning program described their understanding of and commitment to social justice, multicultural competence, and civic engagement” (Einfeld and Collins, 2008, p.95).*

*- “This paper attempts to provide higher education faculty with a model that promotes equity and inclusion by engaging students in developing critical consciousness about their country’s social problems” (Ramia and Díaz, 2019, p.1).*

As it can be seen in the previous examples, the university courses employ this powerful S-L approach to teaching and learning as an effective educational tool to develop consciousness, commitment, and responsibility toward inequality and social problems (Billig and Welch, 2004). It demands students and teachers to be prepared and open for a transformative learning experience toward global inequality.

Finally, the aim (m) was to find out the results described in the works under study. Different authors highlighted the significant benefits that SL has on the university students. The experience of integrating the methodology of S-L benefited the students in developing graduate-level skills in each specialized area, which is useful for its professional and academic life. Moreover, it can be seen that these experiences also offered the university students challenging opportunities to understand the importance of developing critical consciousness and social commitment to fight inequalities.

Nonetheless, there are certainly some challenges while implementing this methodology, as we found one article that shows examples drawn from case study that illustrate the oppressive effects of international service learning (ISL) practices as they do not prepare students to deal with difference and otherness (Larkin, 2015). Despite of this fact, almost all the university students of the papers analyzed reported personal development as an outcome of these S-L experiences. They showed the inherent relationship between the experiences of S-L and the aspects related to inequality and diversity. They also indicated their motivation to learn from the challenges and develop the appropriate skills to overcome them. In the light of these results, it could be said that the benefits far outweigh the challenges.

## Conclusion

This article has explored the literature related to inequality through S-L in higher education through a bibliometric review. The results achieved in this review allowed us to study the topic on two of the most well-known and prestigious databases, such as WoS and Scopus. The present bibliometric analysis was valuable for defining the researchers in the field by measuring the diachronic and personal productivity of the authors. Likewise, it helped to define research work and reference sources by measuring the indicators of dispersion and the impact indicators from a quantitative perspective. From a qualitative perspective, this article also pretended to present the results of the research taking into account the five aspects presented above, namely, type of study, research instruments, sample, objectives, and results of the works under analysis.

Thus, this study shows that there has been an exponential growth in the research topic under study, occurring with the highest scientific productivity in 2012 in both databases. Regarding personal productivity, only a small proportion of authors is responsible for most of the scientific works. The author Tharp, D.S.ı, who appears in both databases, is the one with the highest number of publications. Other authors with matching references in both databases are DuPuis, N., Fickey, A., Grabbatin, B. Hubbard, F.G., and Sterett, S.

In relation to the indicators of dispersion, the study also indicates that there are a total of 13 journals and 16 references distributed in four areas. The journals that coincide in both databases are *Administration Society*, *Journal of Geography*, and *Journal of Transformative Education*. Regarding the language variable, English is the only language used in the scientific production.

The impact indicators show that the highest percentage of references belonged to scientific articles in both databases. In relation to the internationalization of the research, it can be noted that the countries most researched in inequality through S-L in higher education are the United States, followed by Canada, Spain, England, and Ecuador. Moreover, the affiliation with the highest number of authors in both databases among the 15 institutions is the University of Kentucky.

This study shows the growth path of the methodology of S-L in higher education literature, its interdisciplinary approach, and the quality of journals and scholars who have participated in this topic. Moreover, it illustrates that due to the flexibility of the S-L method in assessing the learning process and outcomes, most of the papers are within the qualitative paradigm and the authors tend to use qualitative research instruments such as weekly journals or interviews.

The objectives set in the different papers under analysis revolve around the discussion and reflection on the S-L experiences of students from different disciplines. This interdisciplinary approach can be clearly seen in the wide range of areas in which the SL is implemented. Regarding the results, even though some of the papers state that S-L does not prepare students to deal with difference and otherness, most of the authors reported this approach as a powerful tool to teaching and learning and to develop consciousness, commitment, and responsibility toward inequality and social problems.

In conclusion, this study has explored the role of the S-L methodology on inequality in higher education institutions. The connection between inequality and S-L is so strong that it can be considered as one of the principles for effective practice in S-L at this stage (Billig, 2017). This research has shown that S-L initiatives incorporating aspects related to inequalities also enhance knowledge of and appreciation for diversity with potential for expanding reflections of participants on their own social identities.

These results can help professors, researchers, university students, and educational policy makers in higher education to understand that this method has a significant role not only in higher education institutions but also in society. The limitations are those inherent to bibliometric studies due to possible biases in the selection of search descriptors in the databases and the selection of the documents. This aspect can certainly be used as an element of improvement for a future line of research.

## Data Availability Statement

The original contributions presented in the study are included in the article/Supplementary Material, further inquiries can be directed to the corresponding author.

## Author Contributions

NM-H and SC-R contributed to the design and implementation of the research and performed the revision. GG-G and MS-M verified the analytical methods and supervised the findings of this work. All authors conceived the presented idea, discussed the results, and contributed to the final manuscript.

## Conflict of Interest

The authors declare that the research was conducted in the absence of any commercial or financial relationships that could be construed as a potential conflict of interest.

## Publisher’s Note

All claims expressed in this article are solely those of the authors and do not necessarily represent those of their affiliated organizations, or those of the publisher, the editors and the reviewers. Any product that may be evaluated in this article, or claim that may be made by its manufacturer, is not guaranteed or endorsed by the publisher.
