# Supplementary material for: Exploring Inequality Through Service Learning in Higher Education: A Bibliometric Review Study
Source: Front Psychol. 2022 Mar 11;13:826341. doi: 10.3389/fpsyg.2022.826341 (PMC8963505; doi:10.3389/fpsyg.2022.826341)
Supplement: Supplementary file 1 [file Table_1.docx]

**Supplementary Table 1**

The most relevant information of the contributions in WoS and Scopus

| Title | Authors | Objectives | Methodology | Sample & Research Instruments | | Results |
| --- | --- | --- | --- | --- | --- | --- |
| Sustainable higher education teaching approaches | Krogman, N.T., Bergstrom, A. | To focus on several key concepts and teaching approaches that can engage students in sustainability challenges and give them some of the necessary knowledge and tools to become thoughtful leaders and followers, problem-solvers, and active citizens.  To describe the teaching approaches that help students understand how people, problems, and ecological conditions are interconnected and encourage them to move from individual to collective approaches to sustainability. | Theoretical review | | Not applicable | The chapter explored some of the key teaching approaches that can help student learn about and engage in sustainability, including place-based experiential learning, project- or problem-based learning, case study conflict studies, collaborative learning, social learning, and community service learning. Students will more likely engage with sustainability if they can work on real-world challenges, examine issues in a particular place, struggle with the need to try and understand the problem from multiple vantage points, and draw upon successes elsewhere in process and outcome. |
| Administrative Law and Service Learning: Clients, Repetition, and Race | Sterett, S., DuPuis, N., Hubbard, F.G. | To reflect on a case study of service learning in a course on the legal environment for public administration.  To discuss if service project fits well with public administration teaching and learning, and with social scientific analyses of what law does. | Qualitative: case study | | Administrative law students | The experience of integrating the methodology of service learning in an administrative law class benefited the students in developing graduate-level  research and project management skills. Through participation in the service project, students learned how to synthesize evidence to make it relevant to a client’s needs. This paper shows that service projects offer undergraduate and graduate students intellectually challenging opportunities, useful in professional and academic life. |
| Close encounters of the other kind: Ethical relationship formation and international service-learning education | Larkin, A. | To address International Service Learning (ISL) as a model for engaging students in education for global social justice, including poverty, homelessness or environmental degradation.  To present examples drawn from case studies to illustrate the oppressive effects of ISL practices that do not prepare students to deal with difference and otherness. | Qualitative: case study methodology | | Key partners from different local organizations in a small city in north-western Tanzania  Close participant observation and conducted semi-structured interviews | The results of this paper show that students in this programme were not prepared to engage with the implications of difference or to have their identity challenged in a way that disrupted their identities or sense of purpose. The awareness of socio-economic privilege, widened the gap between ISL participants and host community, obscuring other potential outcomes. |
| Service-Learning as a Methodology to Promote Equity and Inclusion: Best Practice Experience in Ecuador | Ramia, N., Diaz, K | To provide higher education faculty with a model that promotes equity and inclusion by engaging students in developing critical consciousness about their country’s social problems. | Quantitative and qualitative study | | Students from a private liberal arts university in Quito, Ecuador  Civic Attitudes and Skills Questionnaire | This study shows a significant impact of the course on students’ skills has been found on almost all factors in two studies conducted in recent years. |
| Reinforcing Paternalism? The Need for a Social Justice Approach to Prepare Students for Community Engagement at Universities Technology | Grobbelaar, H., Napier, C., Maistry, S. | To discuss the findings of a research study conducted to develop a Community Engagement preparation programme for a group of Universities of Technology students  To gauge students' understanding of service-learning as a form of CE and the rationale for inclusion of service-learning at UoTs. | Qualitative study | | 48 students in the department of Food and Nutrition Consumer Sciences at the Durban University of Technology (DUT).  Five focus groups were conducted. | Students reported personal development as an outcome, but the majority described the experience as “focus on service” without the attention to deeper social issues of inequality and injustice. The results indicated the need to interrogate the current preparation of students for CE and to adopt practices to foster social responsibility within a social justice paradigm. |
| DISRUPTIVE PRACTICES Advancing Social Justice Through Feminist Community Based Service-Learning in Higher Education | Catlett, B. S., Proweller, A. | To address the ways in which feminist-informed community-based service-learning experiences can be a vehicle for advancing social justice. | Qualitative study | | Two different student samples: the first sample consisted of 15 undergraduate students between the ages of 18 and 22 participating in the TBTH program for eight months during the 2009-2010 academic year.  The second sample consisted of 8 undergraduate students between the ages of 18 and 27 participating in the TBTH program for eight months during the 2013-2014 academic year.  In-depth interview questions | In general, the research findings from this empirical study demonstrate how feminist-informed interrogation of structures of privilege and oppression is "existentially disturbing" (Butin, 2010, p. 20) for many students, a process that led them to see and more fully understand the structural and systemic nature of power, from which point they could put their newly emergent awareness to work to create social change. The student narratives coalesce around three salient themes to illustrate this process: (1) Student interrogation of privilege; (2) Positioning interpersonal violence within interlocking systems of privilege, oppression, and inequality; and (3) Moving toward transformation and advancing social justice. |
| Preparing Future Primary Teachers for Society's Challenges: Introducing Service-Learning Methodology within the BSC. in Education | Pinto, A; Baena, V; Mattera, M | To foster the learners’ social and cultural competences, as well as to enhance social consciousness. In addition, there were three secondary objectives, linked to the concept of inclusive education:  • To make the undergraduates familiar with a multi-cultural society  • To fight sexual discrimination, telling future teachers how to work on coeducation and equal  opportunities for men and women with their pupils.  • To remember the importance of transmitting such values to children from an early age. | Qualitative study | | One group of nine pupils from the subjects Library and Attention to Diversity.  Three types of evaluation: one by the teachers (heteroevaluation), one by the students (self-assessment), and one by the NGO’s  voluntary workers.  . | It can be concluded that the Service-Learning project was a complete failure: only one third of the pupils involved really evidenced a higher interest and compromise than the rest of their mates. Nevertheless, this conclusion loses strength when we  listen to the learners’ testimonies: they all appreciated the fact that we wanted them to experience learning in real life, instead of turning them into passive learners of educational theories. In addition, they admitted that it is very positive to train future teachers in the transmission of values to children,  especially concepts related to tolerance, equality, and above all fighting any kind of discrimination. |
| A Proposed Workshop Curriculum for Students to Responsibly Engage Cultural Conflict in Community-Based Service Learning | Tharp, D. S. | To pilot a proposed workshop curriculum to encourage student reflection on social identity and related values and beliefs specific to race and class to address concerns of reinforcing stereotypes and prejudice. | Quantitative study | | 127 students  A short internally created survey was created with seven-point Likert scale questions | The results from the pre-test/post-tests seem to indicate that this pilot workshop curriculum is effective at positively impacting critical reflection, student awareness of culturally-based values, and awareness of when values are being imposed upon others which are all important for service-learning and necessary components of emancipatory learning through transformative education. |
| Service-Learning: Critical Traditions and Geographic Pedagogy | Grabbatin, B., Fickey, A. | This paper has three main aims:  To discuss how service-learning gives geographers the opportunity to demonstrate the practical and political implications of collaborative research methodologies, while conveying powerful conceptual understandings of inequality.  To question the philosophical overlap between experiential and service-based learning in the educational philosophy of John Dewey.  To present a reflection on experiences that implement similar pedagogical projects. | Qualitative study | | The students in  the department’s Appalachian geography course.  Students conducted  interviews with regional leaders | This paper concludes that for geography students, service-learning creates an excellent opportunity  to apply technical skills and to develop an awareness of how they can participate in meaningful change.  For geography instructors, it offers  an opportunity to build long-term relationships with surrounding neighborhoods and organizations, extending the learning community beyond the boundaries of campus  through place-based pedagogy in both a local and international  contexts. |
| The relationships between service-learning, social justice, multicultural competence, and civic engagement | Einfeld, A., Collins, D. | To examine how participants in a long-term service-learning program described their understanding of and commitment to social justice, multicultural competence, and civic engagement. | Qualitative study: constructivist theoretical perspective. | | Ten participants were selected from a university sponsored  AmeriCorps program  Semi-structured interviews | The findings from this study indicate that participating in a long-term service commitment often facilitates the development of multicultural competence in participants. Moreover, the findings from this study reveal the tremendous value of developing relationships across cultural lines. Relationships have the power to prove stereotypes and prejudice wrong and to enable reconciliation. Educators should put students in situations  where the students are able to develop positive relationships cross culturally through sustained contact with people of other cultures. |
| What History is good for Service-learning and studying the past | Smith, M. | To discuss how service-learning in history lead to more active citizenship. To examine how service-learning leads to a deeper appreciation of an historical perspective as a key ingredient for being an engaged citizen. | Qualitative research, based on evidence from a history honors course | | Sixteen students at a private liberal arts college in the northeastern United States.  The sources for their reflections were weekly journals and a  final portfolio essay | This paper shows that studying history with a service-learning component illuminates the presence of the past more dramatically than conventional classroom experiences can, helping students understand historical causation and develop empathetic responses to social inequality. |
